# Severe asymptomatic hypokalemia associated with prolonged licorice ingestion: A case report

**DOI:** 10.1097/MD.0000000000021094

**Published:** 2020-07-24

**Authors:** Young Eun Kwon, Dong-Jin Oh, Hye Min Choi

**Affiliations:** Department of Internal Medicine, Myongji Hospital, Hanyang University College of Medicine, Deokyang-gu, Goyang-si, South Korea.

**Keywords:** asymptomatic, glycyrrhizic acid, hypokalemia, licorice

## Abstract

**Rationale:**

Excessive ingestion of licorice can cause pseudohyperaldosteronism. A few case reports in the available literature have described significant hypokalemia secondary to licorice consumption with clinical manifestations of muscle weakness, paralysis, or severe hypertension. To our knowledge, no report has discussed severe asymptomatic hypokalemia associated with licorice consumption.

**Patient Concerns:**

A 79-year-old man presented to the urology clinic with a several-month history of urinary frequency and a weak stream. Routine laboratory investigations revealed serum potassium (K^+^) level of 1.8 mmol/L, and he was immediately admitted to the nephrology department.

**Diagnoses:**

He was in a good state of health, and systemic and neurological examinations were unremarkable. However, laboratory investigations revealed severe hypokalemia and metabolic alkalosis accompanied with renal K^+^ wasting and hypertension, suggesting a state of mineralocorticoid excess. Hormonal studies revealed low serum renin and aldosterone but normal serum cortisol levels. Detailed history taking revealed that he had used licorice tea daily during the preceding 18 months.

**Interventions and outcome:**

The patient's serum K^+^ returned to normal levels after vigorous K^+^ replacement and discontinuation of licorice intake. He was also diagnosed with benign prostatic hyperplasia during hospitalization and was treated.

**Lessons:**

Chronic licorice ingestion can precipitate severe hypokalemia, although patients may remain asymptomatic. This case report indicates that the severity of a patient's clinical presentation depends on individual susceptibility, as well as the dose and duration of licorice intake.

## 1 Introduction

Excessive ingestion of licorice is known to cause a state of mineralocorticoid excess.^[1]^ However, licorice-induced hypokalemia usually manifests with mild clinical features and a severe presentation is rare. A few case reports in the available literature have described significant hypokalemia secondary to excessive licorice consumption, with clinical manifestations of muscle weakness, paralysis, or severe hypertension. However, to our knowledge, no study has reported severe licorice-induced hypokalemia in asymptomatic patients.

We describe an elderly man who was incidentally diagnosed with significant hypokalemia (serum potassium [K+] 1.8 mmol/L) following prolonged licorice ingestion, without any typical clinical features, such as muscle weakness or electrocardiographic changes.

## 2 Case report

A 79-year-old man presented to the urology clinic with a several-month history of urinary frequency and a weak stream. On presentation, laboratory investigations revealed a serum potassium (K^+^) level of 1.8 mmol/L, and he was immediately admitted to the nephrology department.

He reported a 10-year history of hypertension and dyslipidemia, and received once-a-day telmisartan (40 mg), amlodipine (5 mg), and atorvastatin (10 mg). He reported a history of 3-day hospitalization for acute small intracerebral hemorrhage in November 2017, and his serum electrolyte levels were normal at that time. He denied the use of tobacco or other drugs, including diuretics, laxatives, and herbal medicines, and admitted to the rare use of ethanol. He also denied vomiting, constipation, diarrhea, polyuria, or neurological symptoms.

On initial physical examination, the patient's blood pressure (BP) was 153/85 mmHg, heart rate 71 beats/min, and body temperature 36.9°C. He was in a good state of health, and systemic and neurological examinations were unremarkable. Electrocardiography (ECG) revealed normal sinus rhythm with no abnormal T or U waves. Laboratory investigations were remarkable for hypokalemia accompanied by metabolic alkalosis and mild hypernatremia.

Laboratory tests after admission revealed the following results: serum K^+^ 2.0 mmol/L, sodium 146 mmol/L, chloride 93 mmol/L, total carbon dioxide >40 mmol/L, serum osmolality 302 mOsm/kgH_2_O, random serum glucose 91 mg/dL, blood urea nitrogen 16 mg/dL, serum creatinine 0.6 mg/dL, total serum calcium 9.3 mg/dL, serum phosphorus 3.0 mg/dL, and serum uric acid 4.4 mg/dL. Serum magnesium levels, thyroid and liver function tests, and blood cell counts were within the reference ranges.

Urine analysis showed no abnormalities (specific gravity 1.010, pH 8.0, protein-, glucose-, red blood cells 0–2/high-power field [HPF], white blood cells 0–2/HPF), and urine osmolality was 402 mOsm/kg H_2_O. However, urinary excretion of K^+^ was high in the setting of hypokalemia (urinary K^+^ 37.8 mmol/L, 88.3mmol/g Cr, transtubular K^+^ gradient 15.5).

Based on hypokalemia concomitant with renal K+ wasting and hypertension, we suspected a state of mineralocorticoid excess. Further laboratory investigations revealed low serum renin (<0.10 ng/mL/hour) with low aldosterone (1.91 ng/dL) and normal serum cortisol levels.

**Figure 1:**
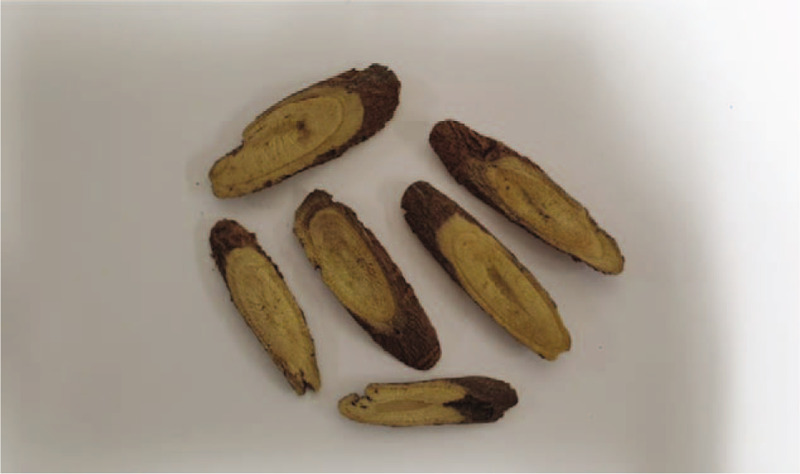
Dried licorice root which was consumed everyday by the patient.

On detailed history taking, the patient admitted to the daily consumption of herbal tea containing licorice since December 2017 (18 months). He was instructed to return with a sample of the product and refrain from further use. We noted that the herbal tea contained 5 jujubes, a small quantity of mugwort, and 20 to 25 g of dried licorice root, which was boiled in 2 L of water. He admitted that he had been consuming this concoction on a daily basis as drinking water (Fig. 1).

He was treated with vigorous K^+^ supplementation and initially received 80 mmol (intravenous) and 6 g (oral) of potassium chloride (KCl) daily. His serum K^+^ level remained at 3.3 mmol/L despite a 7-day course of intensive K^+^ replacement. On the 10th day of hospitalization, his serum K^+^ was restored to a normal level (4.0mmol/L), and BP was 140/82 mmHg. He was also diagnosed with benign prostatic hyperplasia during hospitalization and was treated with silodosin 4 mg and dutasteride 0.5 mg. The patient was discharged after 12 days of hospitalization without any K^+^ supplementation at discharge and was strictly advised to refrain from licorice use. His serum K^+^ level was 4.6 mmol/L at his 1-week follow-up.

The study was approved by the institutional review board of Myongji Hospital. The patient provided informed consent for the publication of his clinical data.

## 3 Discussion

### 3.1 Glycyrrhizic Acid: An Active Ingredient of Licorice

Licorice is widely used as a flavoring agent in food and health products including herbal medicines. Glycyrrhizin is the principal active ingredient in licorice extract, and both licorice extract and glycyrrhizin are approved for use in food items by the US Food and Drug Administration and the World Health Organization (WHO).^[2]^

However, excessive licorice use is known to cause pseudohyperaldosteronism, which is characterized by sodium retention, hypertension, hypokalemia, metabolic alkalosis, and suppression of the renin-angiotension-aldosterone system.

The clinical manifestations of this condition are attributable to glycyrrhizic acid (the active ingredient in licorice), which inhibits a renal enzyme (11 β-hydroxysteroiddehydrogenase [11 β–HSD]). Usually, 11 β-HSD converts bioactive cortisol into inactive cortisone (which cannot bind the mineralocorticoid receptors) within the renal tubules. Glycyrrhizic acid-induced inhibition of 11 β-HSD prevents inactivation of cortisol, which can bind the aldosterone (mineralocorticoid) receptor to produce an aldosterone-like effect.

Similar findings are observed in patients with genetic disorders, such as those with Liddle syndrome and syndrome of apparent mineralocorticoid excess.^[3]^ These conditions were considered less likely etiopathogenetic contributors in our case based on the patient's age and history. Additionally, the patient showed improvement following discontinuation of licorice intake.

Our patient did not specifically reveal a history of licorice intake at the time of initial history taking because in his view, the product was being consumed as his usual tea and not as herbal medicine, and he considered it a safe preparation. Based on our observations in this case, we emphasize the importance of detailed history taking for accurate diagnosis.

### 3.2 Clinical presentation

The severity of the clinical presentation in hypokalemia depends on the degree and duration of low serum K^+^ levels. Patients with mild-to-moderate hypokalemia are usually asymptomatic or may show minor symptoms; however, those with severe hypokalemia (serum K^+^ <2.5 mmol/L) usually present with diverse symptoms.^[4]^ Hypokalemia specifically affects cardiac, skeletal, and intestinal muscles, and patients present with muscle weakness, paralysis, constipation, rhabdomyolysis, cardiac arrhythmia (occasionally fatal), and heart failure.^[3]^

Licorice consumption is rarely associated with serum K^+^ levels <2.0 mmol/L.^[5]^ A few studies have reported severe hypokalemia secondary to licorice intake, and to our knowledge, all patients have presented with clinical symptoms. A review of previous clinical reports revealed that patients with excessive licorice ingestion commonly present with severe hypertension including hypertensive encephalopathy,^[6–9]^ myopathy including paralysis,^[5,10,11]^ and fatal cardiac arrhythmias.^[12,13]^

Serum K^+^ levels were observed to be 2.07 to 3.48 mmol/L in patients with hypertensive urgency and emergencies,^[6–9]^ 2.8 mmol/L in patients with muscle weakness,^[14]^ and 1.7 to 1.8 mmol/L in those with paralysis (Table 1).^[5,10,11]^ A patient with torsades de pointes presented with a serum K^+^ level of 2.4 mmol/L,^[13]^ and a patient with long QT syndrome accompanying cardiac arrest showed a serum K^+^ level of 1.6 mmol/L.^[12]^

**Table 1:**
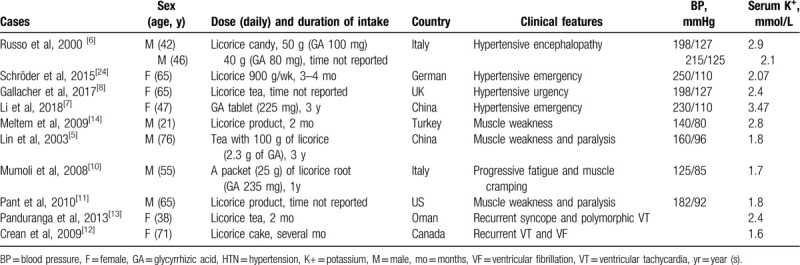
Summary of cases associated with licorice- or glycyrrhizin-induced complications.

Our patient did not show myopathy or ECG changes despite severe hypokalemia and metabolic alkalosis, which could be attributed to a compensatory or adaptive response by the body, whereby patients are usually asymptomatic and present with laboratory abnormalities alone (attributable to chronic renin-aldosterone system activation).

### 3.3 How much is too much?

Bernardi et al^[15]^ performed a study that investigated the action of glycyrrhizic acid in healthy volunteers (4 weeks, n = 24) and observed that the “no observed adverse-effect level” (NOAEL) was 217 mg/day. Higher doses led to sodium retention and lowering of serum renin and aldosterone levels. Based on a study performed in healthy volunteers (8 weeks, n = 40), Bijlsma et al^[16]^ reported that the NOAEL for pure glycyrrhizic acid was 2 mg/kg/day. In 2003, the Scientific Committee on Food reported 100 mg/day (approximately 2 mg/kg) as the safe upper limit for regular ingestion of glycyrrhizic acid.^[17]^ The WHO also states that an intake of 100 mg of glycyrrhizic acid/day would be “unlikely to cause adverse effects in the majority of adults.”^[18]^

The glycyrrhizin content in licorice root varies from 2% to 20%, depending on the type (fresh or dried) and species of licorice and the extraction process.^[1,2,19]^ Schulz et al reported that owing to its mineralocorticoid-like action, the mean daily dose of dried licorice root should not be >5 to 15 g (equivalent to 200–600 mg of glycyrrhizic acid).^[2,20]^

Most previous reports in the available literature have described the ingestion of glycyrrhizin as “licorice product” or “licorice tea” without specifying the type of licorice and the approximate quantity of glycyrrhizin in the licorice product (Table 1).

It is difficult to determine the exact quantity of glycyrrhizin in each type of licorice product. Moreover, the bioavailability of glycyrrhizin in licorice is significantly lower than that of the pure compound.^[2]^ Previous studies that investigated the amount of glycyrrhizin used have reported that 50–575 mg of glycyrrhizin was associated with severe hypertension or paralysis^[6,7,9,10]^ and that a patient who presented with similar features reportedly consumed 2.3 g of this product.^[5]^ The duration of licorice ingestion ranged from 2 weeks to 3 years in previous studies that investigated this topic.^[5,7,10,12,13]^ Relatively short periods of licorice ingestion (several months) were associated with severe arrhythmia.^[12,13]^

A significant individual variation is observed in the susceptibility to glycyrrhizic acid-induced adverse effects.^[6,21]^ Susceptibility to the physiological effects of glycyrrhizin is affected by baseline health status and genetic polymorphisms.^[17,22]^ A partial 11ß-HSD deficiency has been implicated as a contributor in a few cases.^[23]^

Our patient consumed 20 to 25 g/day of dried licorice root, which could be equivalent to approximately 800 to 1000 mg of glycyrrhizic acid daily; this is a relatively large quantity compared with quantities described by previous reports. Interestingly, our patient was asymptomatic despite the consumption of considerable quantities of glycyrrhizin acid and a critically low serum K^+^ level of 1.8 mmol/L.

Our observations in the present case suggest that the severity of a patient's clinical presentation could depend on individual susceptibility, as well as the dose and duration of licorice intake. Licorice is widely used currently, and it is possible that many individuals remain asymptomatic despite significant hypokalemia. Farese et al^[24]^ investigated the role of glycyrrhizic acid as a treatment option in patients undergoing hemodialysis and reported that glycyrrhizic acid supplementation (at a dose of 500 mg/day) led to a moderate reduction in serum K^+^ levels without associated adverse effects.

To date, the clinical significance and outcomes of asymptomatic but severe hypokalemia remain unclear. A literature search did not reveal information regarding severe but asymptomatic hypokalemia regardless of the cause of hypokalemia (eg, excessive licorice use).

### 3.4 Treatment

Licorice-induced mineralocorticoid excess is usually reversible following cessation of licorice ingestion. Recovery usually occurs within days (typically < a week); however, this altered physiological state may persist for several weeks depending upon the quantity of licorice consumed and individual susceptibility.^[1]^ In the present case, discontinuation of licorice consumption and KCl supplementation led to restoration of the patient's serum K^+^ to normal levels in 10 days.

In conclusion, we describe a patient who presented with severe but asymptomatic hypokalemia after prolonged consumption of licorice. Although laboratory investigations revealed sodium retention and K^+^ loss, he did not present with the characteristic presentation of myopathy, severe hypertension, or ECG changes. Our study suggests that the severity of symptoms and clinical features depend on individual susceptibility, as well as the dose and duration of licorice intake in cases of licorice-induced complications.

## Author contributions

**Conceptualization:** Hye Min Choi.

**Investigation:** Hye Min Choi.

**Resources:** Hye Min Choi.

**Supervision:** Dong-Jin Oh, Hye Min Choi.

**Writing – original draft:** Young Eun Kwon.

**Writing – review & editing:** Dong-Jin Oh, Hye Min Choi.
